# Materia Medica

**Published:** 1869-12

**Authors:** 


"MATERIA MEDICA.—This department of the profession is limited to a few vegetable simples, whose medical virtues have been determined by empirical trials, and consists mainly in a few roots, herbs, berries and barks of trees, which operate as emetics, cathartics, stimulants, tonics, etc.; but in order to secure their mystic virtues, they must be administered by a Mis-sis-ke In-nin-e, *i. e.*, literally a medicine man, and as Mis-sis-ke is herbage, grass, etc., it must be understood that these knights of the pestle and pill-box belong to the Botanic faculty.

"All medicines are esteemed equally useless unless their administration is accompanied with the requisite incantations; and these being only known to the mystic fraternity, even the progress of empiricism is deprived of its practical usefulness. The preservation of herbs, etc., is but little attended to; but small quantities are generally used, and these being dried and carefully kept in a leather portmanteau or sack, are commonly secure from humidity or heat. As the greatest amount of sickness prevails in the summer and autumn, it is most convenient for the practitioner to call on nature's drug store for the fresh article.

"PRACTICE OF MEDICINE.—These doctors are commonly very attentive, tender and careful of their patients, of both sexes, and of all ages. In fevers they administer emetics and cathartics; and if from spasm, congestion or other causes, a focus of irritation is suspected, bleeding, cupping and scarifications of the affected part is resorted to. A thin scale of flint is commonly the Indian lancet, scarificator, etc. In cupping, the mouth of the operator, or of some relation to the patient, supplies the place of a cup. In affections of the liver, deranged or impeded functions of the stomach, kidneys, bowels, etc., evacuants, as in fevers, are first resorted to, then recourse is had to epispâstics, such as the inner barks of the butternut tree, or white walnut, also for scarifications, etc.

"*Stopping Blood.*—For this purpose, which grows abundantly on all western prairies, as well as in barren and thinly timbered land, commonly called red root, is chewed or otherwise bruised and applied to the wound. They have many other vegetable styptics and astringents in general use.

"*Decoctions.*—This is a very common form for the administration of remedies, either internally or externally. Bathing affected joints or limbs with a strong decoction of some stimulating or pungent vegetable, is a very popular remedy for rheumatism, white swellings, scrofula, etc.

"*Infusions.*—The bark of the bitter elm, infused in cold water, and drank in considerable quantities, constitutes a popular remedy for the ague and fever.

"*Parturition.*—At such times a small separate lodge or camp is provided for the accommodation of the patient, and a few female attendants, in the centre of which a kind of swing is prepared, fastened above to a cross beam provided for the purpose. In this the patient is suspended by passing the strap or swing under the armpits; in this position the patient, by a slight genuflection, can throw her whole weight upon the swing, or by standing erect upon her feet, thus relieving, in some degree, the tediousness of her painful situation. If, however, the accustomed course of nature is found to be impeded by preternatnral presentation or other causes, an accoucheur is called in to ply the mysteries of his profession upon the patient.

"Here we may be permitted to give a case as an example of Indian skill in this branch of the profession.

"In the summer of 1828, we were called to visit a patient at the distance of about fifty miles. We found her very much prostrated from continued and almost incessant labor, and worse than useless exercises for the last ninety-six hours — a shoulder presentation, with one arm protruding, pulse feeble, with cold perspiration. The doctors (for there were several in attendance) were plying their skill at the top of their medical science. Soon after our arrival we were informed by these professional gentlemen that one more important and often successful operation still remained untried upon the patient, and being assured that she should not be disturbed in the process of the operation, we consented to witness the performance, which was as follows:

"About three or four feet of a grape vine was procured, one end was artfully fashioned with a knife in the likeness of a snake's head, the patient was lying on her back, upon a mattress on the ground; the operator then proceeded silently to pass this artificial serpent over the body of the patient, commencing with the head of the vine snake about the breast, and holding the posterior end in his hand, dexterously imitating the serpentine motions of a snake, and slowly passing it over the abdomen, and towards the feet of the patient. This process was repeated until we deemed it necessary to resort to more efficient means for the relief of the patient. On inquiry we were told by these learned professors, that the fœtus, on seeing the snake approaching it, in that direction, would endeavor to escape from its confinement in order to avoid the dangerous beast, and thus accomplish the object so much desired!

"*Pathology.*—The general tendency of all their arguments and reasoning to explain the pathology and diagnostics of diseases, is to establish the assumed fact, that the patient has fallen under the baleful influence of some supernatural agency."

---

### Book Notices.

*The Transactions of the American Medical Association.* Instituted 1847. Vol. XX. Philadelphia: Printed for the Association. 1869. Pp. 853.

TOGETHER with the Transactions we get a pamphlet, reprinted by order of the Association, being, "The Nomenclature of Diseases. Drawn up by a Joint Committee appointed by the Royal College of Physicians of London. Subject to decennial revision."

The volume of the Transactions for the current year is superior in typographical appearance to many of its predecessors, and contains several articles of permanent value, aside from the formal report of its doings, plan of organization, code of ethics, and catalogue of members. We observe that the names of all permanent members who have not paid their annual dues are omitted. This would clearly be just as to all who became members after the adoption of such a rule, but seems to us unjust to those who were in full membership previously. However, renewed membership is so easily obtained by anybody, that it is hardly worth caviling about.

Several of the papers contained in the volume are of considerable value. Among these we may mention the essays on *Mollities Ossium;* Therapeutic Uses and Abuses of Quinia and its Salts (Dr. Rogers); the Prize Essays on Quinia as a Therapeutic Agent (Dr. Herrick), and on Atrohia and its Salts, by Dr. Bartholow. The paper on Albinism in the Negro Race, by Dr. Jones, of Nashville, is also a very good one.

*The Physician's Hand-Book for* 1870. By William Elmer, M.D., and Albert D. Elmer, M.D. New York: W. A. Townshend & Adams, Publishers, No. 434 Broome Street. 1870.

This hand-book is perhaps too well known to require more than an announcement of its issue. To those who may not have seen it, we say that it contains, in addition to the usual blanks for charges, pocket ledger, etc., some 116 pages of condensed professional information, which must be exceedingly valuable in the way of *suggestions.* It is difficult to conceive of a more compact mass of generally useful information than is here afforded without materially enhancing the cost of a common diary.

